# Editorial on the Research Topic ‘Cell Differentiation, Oxidative Stress, and Oxygen Radicals—In Honor of Prof. Michael Breitenbach’

**DOI:** 10.3390/biom14080920

**Published:** 2024-07-29

**Authors:** Markus Ralser, Mark Rinnerthaler

**Affiliations:** 1 Department of Biochemistry, Charité Universitätsmedizin Berlin, 10117 Berlin, Germany; markus.ralser@charite.de; 2 The Wellcome Centre for Human Genetics, Nuffield Department of Medicine, University of Oxford, Oxford OX3 7BN, UK; 3 Max Planck Institute for Molecular Genetics, 14195 Berlin, Germany; 4 Department of Biosciences and Medical Biology, Paris-Lodron University Salzburg, 5020 Salzburg, Austria

This Special Issue of *Biomolecules* is dedicated to the life and work of our mentor and outstanding scientist, Michael Breitenbach, and marks his 80th birthday, which he celebrated in 2023. As a pioneer in yeast research and a significant contributor to biochemistry and molecular biology, Michael Breitenbach has left an impressive mark on the history of science. As a leitmotif for this Special Issue, we have curated a collection of topics that align with Michael’s diverse scientific interests. Throughout his career, he demonstrated an insatiable curiosity and a willingness to explore a wide array of subjects in great detail and at a depth that escaped others. We aim for this diversity to be reflected in the eclectic range of contributions within this Special Issue.

Born in Mauer (south of Vienna, Austria) in 1943, Michael commenced his studies in chemistry at the University of Vienna in 1962. His formative scientific experiences took root in the laboratory of Professor Otto Hoffmann-Ostenhof, where he studied the process of myo-inositol phosphorylation in the erythrocytes of chickens. This process influenced him profoundly, prompting him to immediately switch to other model systems. As a postdoc, he worked on the structure of bacterial ferredoxin in the research group of Klaus Gersonde (1972–1974). Subsequently, in 1974, he established a working group at Otto Hoffmann-Ostenhof’s institute, focusing on yeast sporulation. Later, as a full professor at the University of Salzburg, Austria, he dedicated himself primarily to unraveling aging processes, using yeast cells as a model system. Yeast has remained a central theme in his research to this day, but he has made seminal contributions to other fields as well.

Michael’s work was pivotal in clarifying several key biological processes. His contributions range from unraveling sporulation-specific metabolic processes, such as inositol phospholipid and dityrosine synthesis, to the study of nonsense suppressor tRNAs, the GTPase Ras2, research on ribosomes (especially the ribosomal protein RPL10), research on apoptosis, the oxidative stress signaling pathways (especially NADPH oxidases), asymmetric inheritance of organelles, and the role of mitochondria in the aging process. Beyond yeast, he made significant contributions to research on allergens, notably cloning and characterizing the allergen Betv1a, the major Birch pollen allergen, for the first time. Without claiming to be exhaustive, publications have also been produced under his aegis that deal with tumor migration or investigate skin diseases, such as epidermolysis bullosa. 

Among the several characteristics that make Michael a special mentor to us, two stand out. The first is his seemingly infinite knowledge, not only about molecular biology, but also about a much broader range of natural sciences, history, culture, and society, to name a few. It is hard to imagine any conversation to which Michael would not be able to add profound insight, typically to a level of detail that would be missed by others. His second unique trait is his unwavering ability to remember what truly matters. In situations where the logic of a scientist collides with our society’s rules, laws, and bureaucracy, his mantra has guided us: “Rules cannot be more important than the people for whom they are made”.

This diversity of scientific interest is also deliberately reflected in this Special Issue, which is contributed to exclusively by long-standing colleagues and collaborators.

Carlo (Bruschi), Valentina (Tosato), and Jason (Sims) have repeatedly worked with Michael [1], addressing questions such as whether aging or oxidative stress can affect genomic stability. An article entitled “**Timing of Chromosome DNA Integration throughout the Yeast Cell Cycle**” [2] was written for this issue, which shows that chromosomal integration of DNA elements is highly cell cycle-specific and can lead to an increase in oxygen radicals.

Nikolaus (Netzer) has been researching cellular changes triggered by hypoxia with Michael [3]. In the article “**Oxidative Stress Reaction to Hypobaric–Hyperoxic Civilian Flight Conditions**” [4], it was shown that oxygen supplementation, according to the recommendations of the Federal Aviation Administration, does not lead to any change in oxidative stress markers in a flight simulation.

Campbell (Gourlay) and Michael have developed a strong interest in the interaction network of ROS (reactive oxygen species) and RAS (rat sarcoma)signaling and their influence on the actin cytoskeleton [5]. In the publication “**Elevated Levels of Mislocalized, Constitutive Ras Signalling Can Drive Quiescence by Uncoupling Cell-Cycle Regulation from Metabolic Homeostasis**” [6], it is shown that the overexpression of constitutively active Ras2 alleles drives yeast cells into quiescence, a process that is mainly initiated by the loss of metabolic control.

Andrey (Kozlov) has been in contact with Michael for quite some time, and has successfully contributed to a Special Issue edited by Michael entitled “Oxidative Stress and Oxygen Radicals” [7]. The article “**Effect of mitoTEMPO on Redox Reactions in Different Body Compartments Upon Endotoxemia in Rats**” [8] was written for the anniversary volume, in which the influence of the mitochondria-targeted antioxidant mitoTEMPO on immune cells and liver cells after an injection of lipopolysaccharides was investigated.

Jing (Li) and Gianni (Liti) have co-authored publications with Michael that deal with mutations and their influence on the nuclear genome and mtDNA, as well as on a population basis [9]. In their paper, “**Spontaneous Mutation Rates and Spectra of Respiratory-Deficient Yeast**” [10], a comprehensive genome-wide screening was used to determine mutation rates and spectra that occur as compensatory reactions in non-respiring yeast strains.

Johannes (Grillari) and Markus (Schosserer), as well as Michael, were part of the National Research Network NFN S93 funded by the Austrian Science Fund (FWF) from 2005 to 2010. Over the years, the three have proven to be not only competent cooperation partners, but also good friends, which has been expressed in many joint publications [11], among other things. For this anthology, senopathies, which are responsible for many aging phenomena, were examined in more detail in the article titled “**Senopathies—Diseases Associated with Cellular Senescence**” [12].

An extremely exciting and personal article was written by Heimo (Breiteneder) and Dietrich (Kraft). The article, “**The History and Science of the Major Birch Pollen Allergen Bet v 1”** [13], describes, in detail, how an ingenious team of scientists isolated, cloned, and characterized the first (pollen) allergen [14].

Shaoping (Li) and Michael share a deep interest in traditional Chinese medicine [15], which has resulted in numerous discussions and joint project proposals. In the article “**Quality Evaluation of Ophiopogon japonicus from Two Authentic Geographical Origins in China Based on Physicochemical and Pharmacological Properties of Their Polysaccharides**” [16], the polysaccharide composition of the dwarf lilyturf was determined.

During his studies in Vienna, Michael not only attended biochemistry, but also seminars in philosophy. A former student and good friend, Gregor (Greslehner) [17], wrote a philosophical article entitled “**Molecular Biology—Pleonasm or Denotation for a Discipline of Its Own? Reflections on the Origins of Molecular Biology and Its Situation Today**” [18], which rounds out Michael’s broad interests.

The two editors, Markus (Ralser) and Mark (Rinnerthaler), supported by the long-term cooperation partners Nana (Grüning) and Nikolaus (Bresgen), would also like to pay tribute to the birthday boy with one publication each. As we have both worked with Michael for many years (and still do) and have produced countless publications together (e.g., [19,20]), we do not want to list common interfaces with Michael (of which there are many). One paper, “**Monogenic Disorders of ROS Production and the Primary Anti-Oxidative Defense**” [21], discusses how a breakdown of cellular redox homeostasis can lead to the onset of human disease, while the other, “**The Janus-Faced Role of Lipid Droplets in Aging: Insights from the Cellular Perspective**” [22], discusses how protein and lipid damage can be detoxified with lipid droplets.
